# Re-evaluating treatment success in trials of peanut oral-immunotherapy: impact of different definitions on efficacy outcomes

**DOI:** 10.1097/ACI.0000000000001077

**Published:** 2025-04-10

**Authors:** Stefanie Berkes, Klara Liddell, Kirsten Beyer, Katharina Blumchen, Antoine Deschildre, Kaarina Kukkonen, Mika J. Mäkelä, Nandinee Patel, Paul J. Turner

**Affiliations:** a National Heart & Lung Institute, Imperial College London, London, UK; b Division Department of Pediatric Respiratory Medicine, Immunology and Critical Care Medicine, Charité Universtãtsmedizin Berlin, Berlin; c Department of Paediatric and Adolescent Medicine, Pneumology, Allergology and Cystic fibrosis, University Hospital Frankfurt, Goethe University, Frankfurt am Main, Germany; d CHU Lille, University Lille, Pediatric Pulmonology and Allergy Department, Hôpital Jeanne de Flandre, Lille, France; e Children's Hospital and Pediatric Research Center, University of Helsinki and Helsinki University Hospital; f Skin and Allergy Hospital, Helsinki University Hospital, University of Helsinki, Helsinki, Finland

**Keywords:** allergen immunotherapy, desensitization, efficacy, food allergy, oral immunotherapy

## Abstract

**Purpose of review:**

Allergen immunotherapy (AIT) is increasingly popular as a treatment strategy for food allergy. Unfortunately, there is significant heterogeneity in reported outcomes, specifically in the dose-thresholds selected for evaluation and the symptoms used to define a “tolerated dose”. These considerations are often investigator-driven and do not consider patient perspectives.

**Recent findings:**

A systematic review by the EAACI CO-FAITH taskforce recently flagged the need to better standardize and harmonize outcomes used in clinical trials of food-AIT. Using less objective symptoms to define dose-limiting symptoms can underestimate the reaction threshold determined at baseline food challenge. As a consequence, this can overestimate the efficacy of food-AIT by 15%. In this review, we perform an individual patient data (IPD) meta-analysis using data from three randomized-controlled trials and one real-world registry, to evaluate how the definition of dose “tolerance” impacts upon reported desensitization rates.

**Summary:**

This analysis provides insight into how clinical efficacy rates for food-AIT are impacted by using different dose thresholds and definitions for when a dose might be consider tolerated. Using more patient-centric outcomes may be a more useful metric to harmonize reporting of outcomes and inform clinical practice, paving the way towards reaching a consensus on outcome reporting in trials of food-AIT.

## INTRODUCTION

The landscape of immunoglobulin E (IgE)-mediated food allergy has dramatically shifted in recent decades. Current data suggests that 2–3% of the global paediatric population have a food allergy [1–3]. Food allergy is the most common cause of potentially life-threatening anaphylaxis in children and adults under the age of 60 years [4]. Accidental reactions are common [5–7]. As a result, food allergy has a significant adverse impact on health-related quality-of-life of both affected individuals and their caregivers. The impact of food allergy extends beyond the individual, affecting industry and government policy and resulting in significant public health burden [8]. A 2013 analysis estimated the annual economic cost of food allergy in the USA at $24.8 billion [9].

Another significant shift has been in the strategies used to manage food allergy. Until recently, this had been restricted to dietary allergen avoidance and provision of rescue medication (e.g., injectable adrenaline [epinephrine]) for treatment of anaphylaxis. Arguably, these are a risk management strategy rather than a treatment. Food allergen immunotherapy (AIT) has become increasingly popular as a disease-modifying treatment. Oral immunotherapy (OIT) for cow's milk and hen's egg is offered routinely in some countries (Spain, Italy) and gaining wider acceptance elsewhere, while a commercial product for peanut-OIT has been approved for clinical use in the USA and Europe [10]. Studies to date have generally determined AIT “success” by an individual's ability to “tolerate” a specified dose at oral food challenge (FC) following treatment [11^▪▪^]. In this review, we summarize the rationale for this, how efficacy outcomes have varied considerably in previous studies and the challenges this causes when counselling food-allergic individuals and their caregivers as to efficacy outcomes and the best form of AIT for any given patient. 

**Box 1:**
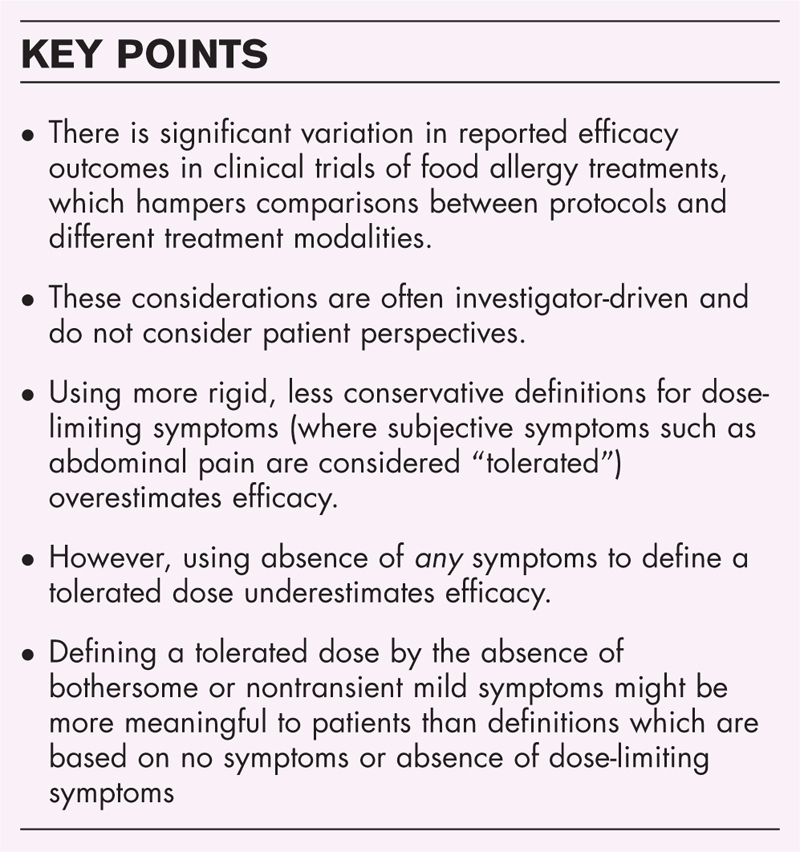
no caption available

## RATIONALE FOR FOOD CHALLENGE AS A SURROGATE MARKER OF EFFICACY

The main aim of food-AIT is to increase the reaction threshold for a food-allergic individual, which in theory would increase the amount that person can be exposed to without reacting [10]. Evidence also suggests that treatment results in reactions of lower severity, where these occur [12]. In some patients, the reduction in risk of accidental exposures may be sufficient [13], however for others, the aim is to achieve a degree of dietary normalization or even ability to eat the allergen ad libitum [14]. Unfortunately, achieving the latter (something often described as “tolerance”) is uncommon, and thus food-AIT cannot at this time be considered a curative therapy [10]. If the primary aim is to reduce risk of accidental reactions, then the most relevant efficacy outcome should be a reduction in accidental reactions, something which has been described as “field efficacy”. The United States Food and Drug Administration (FDA) defines this as “the rate and/or severity of reactions to food exposures encountered outside a controlled clinical setting” [15]. However, the risk of accidental reactions is dependent on exposure: different patients follow different levels of dietary avoidance – something which may well change following treatment. To assess rate of accidental reactions pre and post-AIT would require an initial observation period of at least 2–3 years (in order to capture sufficient events) and then a similar period of monitoring after treatment. Data capture and correcting for confounding by changes in dietary avoidance would be challenging. All these factors mean that using rate of accidental reactions as an outcome measure is not feasible in clinical trials [16].

To address these issues, both the FDA and European Medicines Agency (EMA) have accepted the use of a double-blind, placebo-controlled food challenge (DBPCFC) as a surrogate marker of efficacy in trials of food-AIT [15–18]. The FC can provide evidence of both an increase in reaction threshold and a decrease in symptom severity to any given level of exposure, in a controlled setting. However, such outcomes are not necessarily considered patient-centric: while adopting shared decision-making has rightly become a standard for consenting patients to food-AIT, efficacy data (“tolerance to 600 mg food protein”) does not always translate into clear and tangible patient benefits [19]. Studies have sought to overcome this by reporting changes in health-related quality-of-life (HRQL), usually by parent proxy (especially in children under 12 years) [11^▪▪^]; however, the inclusion of patient/caregiver perspectives remains insufficient, and this is a high-priority gap [20]. Indeed, some large clinical trials have failed to demonstrate consistent evidence of improved HRQL with peanut-OIT across age groups [21,22]. This has resulted in some health-economic assessments recommending against implementation of OIT in routine practice [23,24].

## WHAT EFFICACY OUTCOMES HAVE BEEN USED IN THE LITERATURE?

**FIGURE 1:**
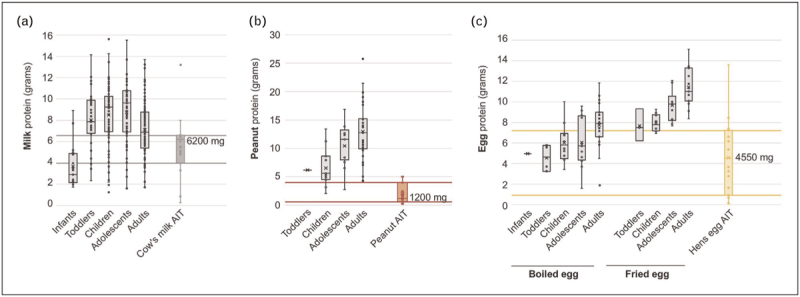
Levels of food protein used to define food challenge thresholds or regular consumption amounts used as primary efficacy outcome in trials of food-AIT for milk (a), peanut (b) and egg (c). Data expressed as median amounts of food protein with interquartile range. Figure reproduced from reference [11^▪▪^] with permission.

Most trials have utilized FC outcomes (such as desensitization or absence of “dose limiting symptoms”) to a specific dose of allergen as the primary outcome (see Fig. 1) [11^▪▪^]. However, there is no consensus as to what that specific allergen dose should be, nor how to define what symptoms are “dose limiting”. This has led to significant heterogeneity in outcomes used [11^▪▪^]. Furthermore, these are investigator-driven outcomes (e.g., informed by international consensus criteria on how to conduct DBPCFC) which do not prioritize patient or caregiver considerations. While other allergic diseases (such as eczema) have established core outcome sets (COS), there is no current COS for food allergy. The EU-funded Core Outcome Measures for Food Allergy (COMFA) initiative aimed to establish a COS for food-AIT trials, but was only partially successful: only two outcomes, “allergic symptoms” and “quality of life” achieved consensus for inclusion as ‘core’ outcomes, with no agreement in terms of efficacy outcomes related to desensitization [25^▪^].

**FIGURE 2:**
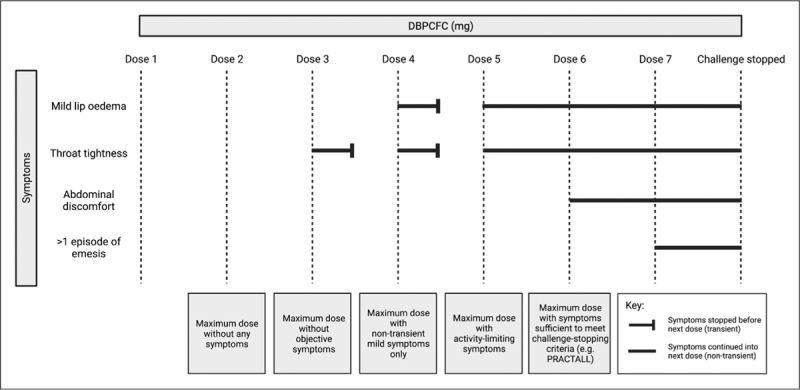
Typical evolution of symptoms during a food challenge. Doses 1 and 2 do not cause any symptoms and are thus fully “tolerated”. Dose 3 causes transient throat tightness. Dose 4 causes transient mild lip oedema and a recurrence of throat tightness – which might be considered “dose-limiting” according to definitions from the Consortium for Food Allergy Research (CoFAR) [31,33] but not PRACTALL [28]. Even with the onset of abdominal discomfort after dose 6, the challenge continues with dose 7 after which the abdominal pain which becomes persistent and thus PRACTALL challenge-stop criteria are met.

The criteria used to define whether a dose is tolerated, that is absence of any “dose limiting symptoms” also varies between studies; indeed, some studies fail to clarify their definition of a “tolerated” dose altogether [26,27]. Changing the symptom-threshold required for a dose to be considered “tolerated” can have a very significant impact on reaction threshold. Figure 2 illustrates this issue – the vast majority of allergic patients do not experience an all-or-nothing response at FC; rather, symptoms evolve from mild and often subjective symptoms (such as oral pruritus) at low levels of allergen exposure. At each subsequent dose, the patient may develop new symptoms until experiencing sufficient features to fulfil predefined challenge-stop criteria.

To reduce inter- and intra-clinician variation in determining challenge-stopping symptoms, many trials have used the 2012 PRACTALL consensus criteria [28] (which have just been recently updated) [29]. Whilst this has added a level of objective reproducibility to determine reaction thresholds, in practice, patients often experience symptoms that cause discomfort before they reach this stage – particularly oropharyngeal and gastrointestinal symptoms (such as throat tightness, nausea and abdominal pain) [29]. Even when PRACTALL is used, interpretation of symptoms is dependent on clinicians’ and patients’ prior experiences [28–30]. Some criteria have therefore required a lower level of objective symptoms to define “dose-limiting” (Table S1, Supplemental Digital Content) [31], but this can adversely impact both objectivity and reproducibility of the DBPCFC as an assessment tool [32]. Indeed, a publication from the Consortium for Food Allergy Research (CoFAR) states that the FDA and CoFAR investigators determined that a previous grading system [33] to describe adverse events associated with procedures specific to food allergy studies (such as oral FCs and OIT dosing) “was too subjective for widescale use,” resulting in the development of a system that incorporates objective signs and symptoms [31]. Using an individual patient data (IPD) meta-analysis, we previously demonstrated that the use of less-objective stopping criteria could potentially over-estimate the efficacy of OIT (as determined by tolerating a specified allergen dose) by 15% [32]. This was predominantly due to the use of less objective symptoms underestimating the baseline reaction threshold, leading to an overestimate of treatment effect.

## IMPACT OF HETEROGENEITY OF OUTCOMES ON HEADLINE RATES OF TREATMENT SUCCESS

To further explore how different definitions for dose-limiting symptoms impact efficacy, we undertook a further IPD meta-analysis using data from three peanut-OIT randomized-controlled trials [34–36] and a registry of patients undergoing peanut-OIT in a real-world setting (Table S2, Supplemental Digital Content). Symptoms during both baseline and post-OIT food challenges were then re-evaluated by 2 independent assessors according to the following definitions:

(1) No dose-limiting symptoms (DLS): Maximum dose at which no dose-limiting symptoms occurred according to PRACTALL or equivalent. Under this definition, symptoms not considered dose-limiting, such as isolated abdominal pain or nausea, or mild skin symptoms, would be considered “tolerated”.
(2) No activity limiting symptoms: The highest dose without any “activity-limiting symptoms” which equates to the dose before the presence of grade 2+ CoFAR symptoms [33].
(3) No nontransient symptoms: The highest dose at which there are no symptoms, except for transient mild (grade 1+ COFAR) symptoms [33]. Under this definition, mild COFAR grade symptoms, such as an itchy mouth or throat, occurring for <20 min would be considered “tolerated”.
(4) No observed adverse event level (NOAEL) (objective): The highest dose before onset of any objective symptoms or signs. Under this definition, any degree of subjective symptoms (including significant abdominal pain) would be considered “tolerated”.
(5) NOAEL (any): Under this definition, any symptoms – irrespective of severity or duration – would be considered “not tolerated”.

Some food-AIT studies have also included the requirement for a minimum 10-fold or even 100-fold increase in maximum tolerated dose to define treatment success [37]. These were therefore included as a composite measure for the first three definitions. Inter-rater agreement in evaluating reaction thresholds using these definitions was also assessed. There was complete agreement for the “NOAEL (any)” threshold (κ = 1.00), and “excellent” agreement for the other definitions used: “No DLS” (κ = 0.97), “No activity-limiting symptoms” (κ = 0.98) “NOAEL (objective)” (κ = 0.97), “No nontransient symptoms” (κ = 0.82).

Results from the analysis are shown in Table 1. The use of more rigid, less conservative definitions for dose-limiting symptoms overestimates efficacy, in that some participants will experience symptoms (such as abdominal discomfort) which are “tolerated” but were they to occur at home, would cause anxiety and probably prompt treatment. Using “no symptoms” as the definition for tolerance might also be expected to over-estimate efficacy, since this under-estimates baseline reaction threshold [32]. However, following AIT, many patients continue to experience mild transient symptoms such as oral pruritus to low doses, thus classifying these low-grade symptoms as “not tolerated” actually underestimates efficacy. Indeed, continuing to experience such symptoms to low levels of exposure can serve as a useful “early warning” against further consumption of a potentially-contaminated food. Therefore, it is probably unreasonable to classify mild transient symptoms as “not tolerated”. In support of this, a survey of 857 patients who had undergone OIT and their parents suggested that experiencing mild symptoms only to the triggering allergen is an acceptable patient-related outcome in the vast majority of respondents [38^▪▪^].

**Table 1:** Impact of using different definitions of “tolerated dose” on reported rates of successful peanut-OIT, assessed per protocol and by intention-to-treat (ITT) analysis

					No DLS (%)		No activity-limiting symptoms (%)		No nontransient symptoms (%)		NOAEL (objective) (%)	NOAEL (any) (%)
Study	Challenge-stopping criteria	Dose threshold (mg)	Reported success (%)		No min. increase	With min. 10-fold	No min. increase	With min. 10-fold	No min. increase	With min. 10-fold	No min. increase	No min. increase
Blumchen *et al.*[34]	Objective clinical symptoms	300	Per-protocol	83 (20/24)	83 (20/24)	71 (17/24)	67 (16/24)	54 (13/24)	63 (15/24)	54 (13/24)	67 (16/24)	54 (13/24)
			ITT	74.2 (23/31)	65 (20/31)	55 (17/31)	52 (16/31)	42 (13/31)	48 (15/31)	42 (13/31)	52 (16/31)	42 (13/31)
BOPI study [35]	PRACTALL symptoms	1443	Per-protocol	100 (38/38)	100 (38/38)	95 (36/38)	92 (35/38)	84 (32/38)	71 (27/38)	71 (27/38)	92 (35/38)	61 (23/38)
			ITT	81 (38/47)	81 (38/47)	77 (36/47)	74 (35/47)	68 (32/47)	57 (27/47)	57 (27/47)	74 (35/47)	49 (23/47)
Deschildre, France	PRACTALL symptoms	2191	Per-protocol	64 (25/39)	64 (25/39)	38 (15/39)	62 (24/39)	33 (13/39)	56 (22/39)	33 (13/39)	59 (23/39)	49 (19/39)
Kukkonen *et al.*[36]	Objective clinical symptoms	1255	Per-protocol	84 (25/30)	93 (28/30)	80 (24/30)	93 (28/30)	80 (24/30)	87 (26/30)	73 (22/30)	90 (27/30)	27 (8/30)
			ITT	85 (33/39)	72 (28/39)	62 (24/39)	72 (28/39)	62 (24/39)	67 (26/39)	56 (22/39)	69 (27/39)	21 (8/39)

**FIGURE 3:**
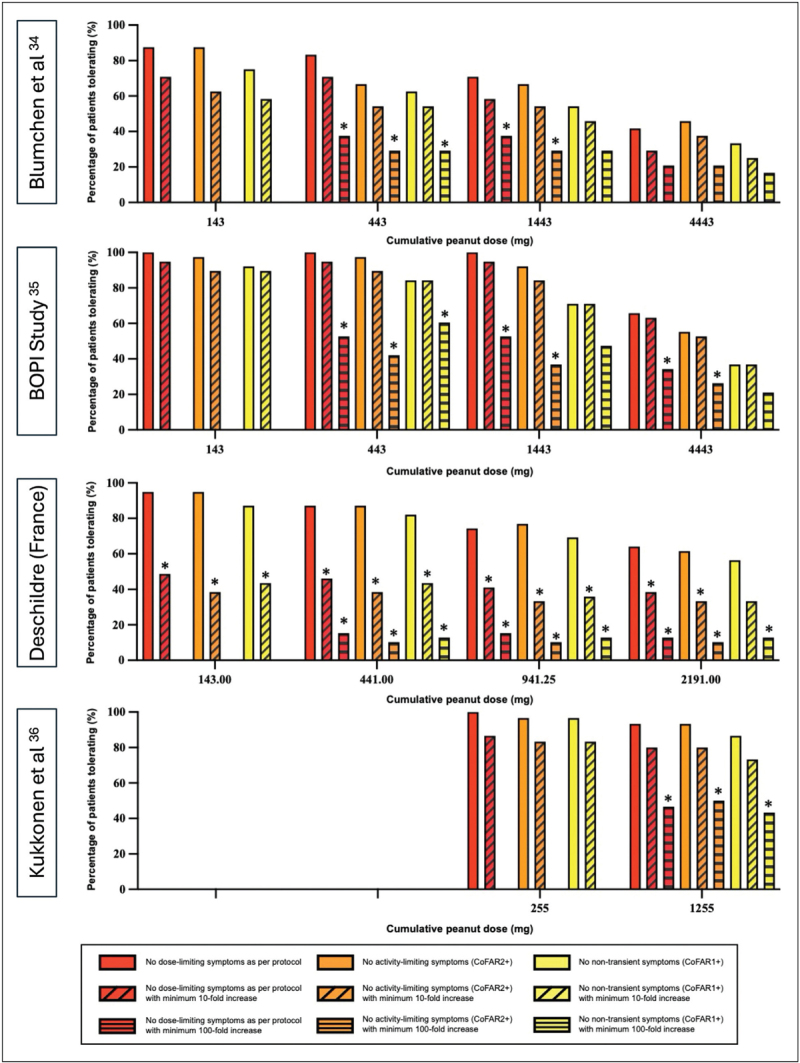
Impact of using a composite efficacy outcome which requires a 10-fold or 100-fold increase in maximum tolerated dose in the four included studies in this IPD-meta-analysis. ∗*P* < 0.05 (Mann–Whitney *U*-test ) comparing the inclusion of a 10- or 100-fold increase to no required minimum.

The analysis also clearly demonstrates that headline efficacy will vary depending on the dose chosen for evaluation of primary outcome: choosing a higher dose confers greater protection, but fewer patients achieve that outcome: particularly if other requirements (such as a minimum 10-fold increase in threshold) are also needed (Fig. 3). The PEPITES trial studied epicutaneous immunotherapy for peanut allergy, and in subjects reacting to ≤3 mg at baseline FC required at least a 100-fold increase in reaction threshold to achieve the primary outcome (reacting to ≥300 mg peanut protein after treatment) [37]. While a 100-fold increase is clearly a much more rigid definition of treatment success (and impossible to demonstrate in those who react to >30 mg since the food challenge does not usually include sufficiently high a top dose), it may be appropriate to have “tolerance to 300 mg peanut protein” (approximately 1½ peanuts) as a primary outcome of efficacy in peanut-allergic individuals who are low-dose reactors.

## INTERPRETING EFFICACY DATA FOR PATIENTS AND THEIR FAMILIES

**FIGURE 4:**
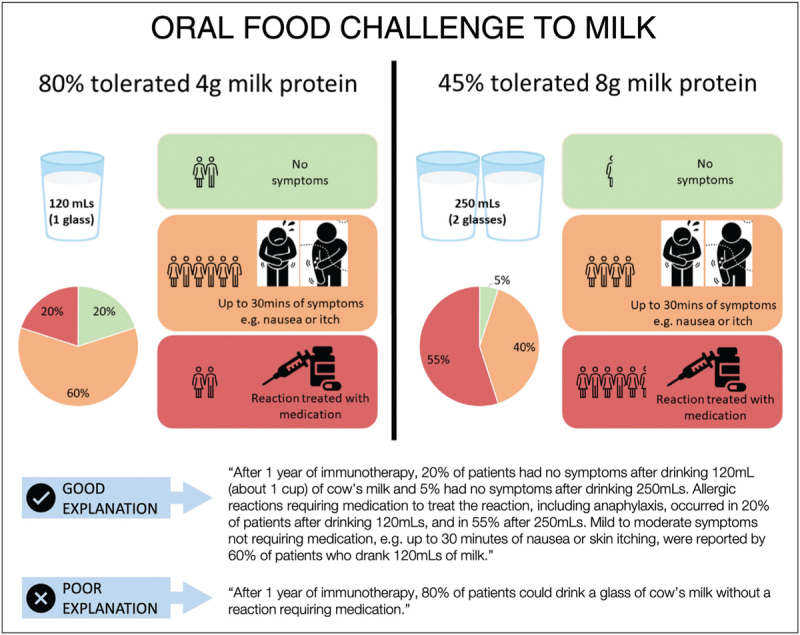
Communicating efficacy outcomes from trials of F-AIT can be done effectively. Figure reproduced from [40] with permission.

Informed consent is a critical and legally-mandated step towards both participation in a clinical trial and/or receiving a clinical intervention. Given that food-AIT is becoming routine, it may no longer be ethical for studies of food-AIT to include a placebo group [39]. Providing a meaningful interpretation of expected clinical efficacy is therefore needed. Historically, this might have involved simply telling patients and their caregivers that the treatment is 80% successful. However, such a statement fails to provide the necessary detail in terms of what “success” means, specifically what amount of the food allergen might be consumed and what symptoms might be experienced. It is possible to communicate this in a format that is understandable by the majority of potential patients and their families (see Fig. 4) [40].

Furthermore, as we move towards different modalities of food-AIT being available (rather than just being limited to OIT), with or without adjunct treatment with a biologic such as anti-IgE therapy [41], it will become increasingly important to compare relative efficacy across different treatment protocols. Unless action is taken now to ensure certain efficacy outcomes are used in all studies, advising patients as to the best treatment option for them will become almost impossible.

## INCORPORATING THE PATIENT VOICE

Understanding what outcomes from food-AIT are meaningful to patients is going to require direct engagement with patient groups. Experience from the COMFA project has demonstrated that this may be challenging [25^▪^]. However, patient/caregiver engagement must be a key step in reaching consensus on what the optimal outcomes for F-AIT interventions should be. To address this, the CO-FAITH taskforce undertook a prospective cross-sectional international study to understand caregiver perspectives on the burden of FA, their mindset towards the treatment, and prioritize potential clinically relevant outcomes for them. 857 patients and/or their caregivers were recruited, all of whom has undergone AIT to either milk (42%), peanut (40%) or egg (19%) [38^▪▪^]. A data-driven analysis demonstrated that patients/caregivers could be categorized into a number of clusters depending on whether their motivation for food-AIT was to achieve “protection from accidental exposures”, “improve socialization” with respect to food consumption, “normalization” (being able to forget about their allergy and no longer needing to carry rescue medication) or a combination of these. Interestingly, while 70% of the cohort felt that an important patient-reported outcome would be eating a small serving of the allergen with no symptoms, this increased to 90% if mild symptoms would be tolerable. This suggests that defining a tolerated dose by the absence of bothersome or nontransient mild symptoms might be more meaningful to patients than definitions which are based on no symptoms (NOAEL) or absence of dose-limiting symptoms such as PRACTALL or CoFAR [33].

## CONCLUSION

To allow patients and their caregivers to truly grasp the potential benefit of food-AIT, they need to understand how treatment success has been defined in clinical trials, including what constitutes a “tolerated dose”, how this translates to “field efficacy”, i.e. real-life protection against unintended allergen exposure – and in turn, how this compares to their expectations and goals for treatment. As we move towards being able to offer a range of different treatment modalities to patients, we need to be able compare efficacy across different studies – something that can only be done if studies report “tolerance” to a specific dose or doses in a consistent way. Effective discussions with patients will also require better understanding of the actual risks of treatment. While this review has focussed on defining treatment “success”, significant heterogeneity also exists across studies in reporting adverse events. Consensus is needed on how best to report both efficacy and safety outcomes, with input from stakeholders including patients/caregivers. Reaching consensus will pave the way for clinicians to help their patients to make truly informed decisions about embarking on treatments for food allergy.

## Acknowledgements

*The authors are grateful to Drs Pablo Rodríguez Del Río, Montserrat Álvaro-Lozano, Montserrat Fernandez Rivas and other colleagues in the EAACI CO-FAITH taskforce.*

### Financial support and sponsorship

*This research was supported by the UK Medical Research Council (MR/K010468/1, MR/W025639/1).*

### Conflicts of interest

*P.J. Turner reports grants from UK Medical Research Council, NIHR/Imperial BRC and JM Charitable Foundation; personal fees from UK Food Standards Agency, outside the submitted work. M.J. Mäkelä from GSK and Orion Pharma, outside the submitted work. K. Blumchen reports institutional grants by Novartis Pharma GmbH, Aimmune Therapeutics, DBV Technologies, and Hipp GmbH; Consulting fees by Novartis Pharma GmbH, Nestle, Bencard Allergie, Aimmune Therapeutics, ALK, and DBV Technologies; Honoraria by Aimmune Therapeutics, DBV Technologies, Novartis Pharma GmbH, Thermo Fisher Scientific, Viatris Healthcare GmbH, Allergopharma, and Danone; Meeting/travel support by DBV Technologies, Novartis Pharma GmbH, and Aimmune Therapeutics; and Participation Data Safety Monitoring Board/Advisory Board by Novartis Pharma GmbH. A. Deschildre has received speaker/consulting fees from Aimmune therapeutics, ALK, AstraZeneca, DBV Technologies, GSK, Nestlé, Regeneron Pharmaceuticals Inc., Sanofi, Stallergenes Greer, Viatris and Celltrion. K. Beyer reports funding to her institution for grants from the German research foundation, the German Federal Ministry of Education and Research (BMBF), the German Federal Ministry of Food and Agriculture (BMEL), Aimmune, Danone, DBV, Hipp, Infectopharm, Hycor, Novartis; and having received speaking honoraria from Aimmune, ALK, Danone, Infectopharm, Nestle, Hycor, ThermoFisher, Kantar Health, Labor 28, Stallergenes, consulting fees from Hipp, Novartis and advisory board honoraria from Aimmune, ALK, Danone, Viatris, Nestle, Allergy Therapeutics, Novartis, Kantar Health, Primus Consulting, Stallergenes outside the submitted work. The other authors have nothing to disclose.*

## Supplementary Material

Supplemental Digital Content

## Supplementary Material

Supplemental Digital Content

## REFERENCES AND RECOMMENDED READING

Papers of particular interest, published within the annual period of review, have been highlighted as:

▪ of special interest
▪▪ of outstanding interest
