# Supplementary material for: Re-evaluating treatment success in trials of peanut oral-immunotherapy: impact of different definitions on efficacy outcomes
Source: Curr Opin Allergy Clin Immunol. 2025 Apr 10;25(3):185–93. doi: 10.1097/ACI.0000000000001077 (PMC12052049; doi:10.1097/ACI.0000000000001077)
Supplement: Supplemental Digital Content [file coaci-25-185-s001.pdf]

	COFAR v3[31]	PRACTALL (2012)[28]	Updated PRACTALL (2024)[29]
Suggested FC stopping criteria:	One red or 2 orange symptoms	One red or 3 orange symptoms	One red or 2 orange symptoms
**SKIN**			
Scratching	Occasional only<br>Protracted	Mild/moderate<br>Continuous with excoriations	Any
Erythema	Faint<br>More than a few areas<br>Pronounced / Generalised	Few faint areas<br><50% of body<br>Generalised / >50%	Few faint areas<br><50% of body<br>Generalised / >50%
Hives/urticaria	Few/localised<br>Numerous	<3 lesions<br>≥3 lesions	1-2 lesions (not perioral or due to contact)<br>≥3 lesions (not perioral or due to contact)
Angioedema	Mild lip<br>Any significant	Mild lip<br>Any significant	Prominent lip or ear oedema<br>Facial or generalized oedema
**UPPER RESPIRATORY**			
Rhinitis	Any	Moderate, frequent<br>Severe, persistent and/or long bursts	Persistent and significant rhinorrhea/sneezing/rhinitis
Eyes	Not specified	Intermittent rubbing<br>Continuous rubbing/reddening	Minimal reddening, rubbing of eyes<br>Conjunctival hyperemia (without rubbing)
**LOWER RESPIRATORY**			
Cough	Occasional<br>Persistent	>3 discrete episodes of throat clearing<br>Frequent dry cough	Intermittent cough with throat clearing<br>Frequent cough without resp. compromise<br>Cough with respiratory compromise
Objective wheeze	Any	Any	Any
**PHARYNGEAL/LARYNGEAL**			
Oral cavity	Not specified	Itchy mouth	Itchy mouth
Throat	Discomfort<br>Tightness<br>Hoarseness/stridor	Itchy throat<br>Persistent tightness or pain<br>Hoarseness/stridor	Itchy throat, intermittent throat clearing<br>Persistent tightness or pain<br>Non-transient hoarseness/stridor
**GASTROINTESTINAL**			
Abdominal discomfort	Mild nausea/pain ± ↓activity<br>Persistent moderate nausea or pain with ↓activity	Mild nausea/pain ± ↓activity<br>Moderate nausea/pain ± ↓activity<br>Severe nausea/pain ± ↓activity	(Any) nausea or mild abdominal pain<br>Persistent non-distractable abdominal pain<br>Persistent severe abdominal pain
Vomiting	Vomit due to gag<br>Any other vomit	1 episode<br>2+ episodes	Vomit due to gag or taste aversion<br>Any other vomit
Diarrhea	Not specified	1 episode 2+ episodes	1 episode 2+ episodes
**CARDIOVASCULAR/NEUROLOGICAL**			
	Clinically significant hypotension<br>Change in mental status	Weak/dizzy/tachycardia<br>Hypotension<br>Change in mental status / LOC	Feeling weak, tired, upset/agitated<br>Clinically significant hypotension<br>Significant change in cognition / LOC

Table S1: Comparison of challenge-stop criteria used in the literature for clinical trials of food allergy. Any **red** symptom is generally considered to be dose-limiting, while the different schemata require a differing number of **orange** symptoms which imply a likely allergic reaction. **Green** symptoms do not contribute towards the decision to terminate a food challenge.
