# Supplementary material for: Re-evaluating treatment success in trials of peanut oral-immunotherapy: impact of different definitions on efficacy outcomes
Source: Curr Opin Allergy Clin Immunol. 2025 Apr 10;25(3):185–93. doi: 10.1097/ACI.0000000000001077 (PMC12052049; doi:10.1097/ACI.0000000000001077)
Supplement: Supplemental Digital Content [file coaci-25-185-s002.pdf]

STUDY	POPULATION	INTERVENTION	ORAL FOOD CHALLENGE DETAILS
Blumchen et al.[34]	Multicentre RCT in Germany<br>n=62 (31 active / 31 control)<br><br>3-17 years (38 male / 26 female)<br><br>**Eligibility criteria:**<br>Serum peanut specific IgE >0.35kUA/L and challenge-proven clinically relevant PN allergy.	1:1 randomisation<br><br>**Active:**<br>Chocolate pudding containing peanut flour. Biweekly up-dosing, lasting a maximum of 14 months<br><br>Maintenance dose determined by initial eliciting dose and maintenance phase lasted for 8 weeks<br><br>**Control:**<br>Placebo of chocolate pudding vehicle without PN flour (blinded).	**Design:**<br>• Open FC (over 3 days)<br>**Dosing schedule:**<br>• Day 1: 3, 10, 30, 100mg; Day 2: 100, 300, 1000, 3000mg; Day 3: 4500mg PN protein.<br>**Time interval between doses:**<br>• 120 minutes<br>**Criteria for stopping:**<br>• Objective clinical symptoms<br>**Matrix – vehicle:**<br>• Boiled apple sauce + crushed roasted PN<br>**Clinical efficacy end point:**<br>• No objective symptoms to 300mg PN protein
BOPI Study[35]	Single centre RCT in United Kingdom<br>n=47 (32 active / 15 control)<br><br>8-16 years (27 male / 20 female)<br><br>**Eligibility criteria:**<br>Clinical reaction to <1.44g PN protein at baseline FC	2:1 randomisation<br><br>**Active:**<br>Updosing with boiled PN for ~6 months prior to transition on to roasted PN to a total of 1 year.<br><br>Maintenance dose: 800mg PN protein.<br><br>**Control:**<br>Peanut avoidance	**Design:**<br>• DBPCFC (2 visits, more than 7 days apart)<br>**Dosing schedule:**<br>• 3, 10, 30, 100, 300, 1000, 3000mg PN protein<br>**Time interval between doses:**<br>• 30-minutes<br>**Criteria for stopping:**<br>• PRACTALL<br>**Matrix – vehicle:**<br>• Soya-butter sandwich +/- roasted peanut flour<br>**Clinical efficacy end point:**<br>• No symptoms to cumulative 1443mg peanut protein that met PRACTALL challenge-stop.
Deschildre, France	Single centre, prospective cohort stdy in France<br>n=62 (62 active)<br><br>3-17 years (39 male / 23 female)<br><br>**Eligibility criteria:**<br>Children with a challenge-proven peanut allergy, able to tolerate at ≥5mg peanut protein.	Allocation not applicable<br><br>**Active:**<br>Starting dose of 2mg peanut protein with an apple sauce vehicle<br><br>Build-up phase (7 doses) up to 300/400mg peanut protein flour (2 doses), then “Curly” snack (3 doses) and then whole peanuts (2 doses: first dose, 1 peanut and second dose, 2 peanuts)<br><br>Maintenance dose: 300/400mg	**Design:**<br>• Open FC<br>**Dosing schedule:**<br>• (Optional: 0.25), 1.25, 2.5, 12.5, 50, 125, 250, 500, 1250mg, (optional: additional 1250mg)<br>**Time interval between doses:**<br>• 20-30 minutes<br>**Criteria for stopping:**<br>• PRACTALL<br>**Matrix – vehicle:**<br>• Apple sauce + crushed roasted peanut<br>**Clinical efficacy end point:**<br>• Full challenge without any symptoms OR only mild symptoms that do not require treatment
Kukkonen et al.[36]	Single centre RCT in Finland<br>n=60 (39 active / 21 control)<br><br>6-18 years (35 male / 25 female)<br><br>**Eligibility criteria:**<br>Moderate-to-severe reaction at baseline DBPCFC to peanut.	Allocation to treatment or control based on the preferences of patients<br><br>**Active:**<br>Starting dose of 0.1 mg peanut protein in a margarine matrix<br>Weekly to biweekly updosing, lasting 8 months<br>Maintenance dose: 4 peanuts<br><br>**Control:**<br>Peanut avoidance.	**Design:**<br>• DBPCFC (2 non-consecutive visits)<br>**Dosing schedule:**<br>• 5, 50, 200, 1000mg<br>**Time interval between doses:**<br>• 30-minutes<br>**Criteria for stopping:**<br>• Objective clinical symptoms<br>**Matrix – vehicle:**<br>• Margarine +/- defatted roasted peanut flour<br>**Clinical efficacy end point:**<br>• Tolerance to 5000mg peanut protein

Table S2: Studies included in the IPD-meta-analysis
